# Supplementary material for: Anaerobic Feces Processing for Fecal Microbiota Transplantation Improves Viability of Obligate Anaerobes
Source: Microorganisms. 2023 Sep 5;11(9):2238. doi: 10.3390/microorganisms11092238 (PMC10535047; doi:10.3390/microorganisms11092238)
Supplement: Supplementary file 1 [file microorganisms-11-02238-s001.zip › Supplementary Tables S1-S3.pdf]

Supplementary tables

Sample	Volunteer	Sexe	Age at donation	Comorbidities	Specifics	Storage time [a]
#1	1	Female	26	None	Volunteer	2 years
#2	2	Female	28	None	Volunteer	2 years
#3	3	Female	28	None	Volunteer	2 years
#4	4	Female	29	None	Screened FMT donor	2 years
#5	5	Male	26	Diabetes Mellitus type 1	Volunteer	1 month
#6	6	Male	23	None	Volunteer	1 month
#7	1	Female	28	None	Volunteer	1 month
#8	2	Female	30	None	Volunteer	1 month

Supplementary Table S1. Demographics of volunteers.

[a] At time of culturing experiment with CFU quantification.

Supplementary Table S2. Colony forming units (CFU) per gram of feces per volunteer and culturing condition.

Sample	Storage time	Condition	Cultured bacteria (CFU/g feces)		
			Aerobic	Partial anaerobic	Full anaerobic
1	2 years	AN_0	2,68E+07	1,39E+10	2,37E+10
		$AN_{2.5}$	2,24E+07	1,33E+10	1,91E+10
		AE_0	5,76E+07	1,63e+10	1,94E+10
		$AE_{2.5}$	5,60E+07	1,61E+10	1,69E+10
2	2 years	AN_0	1,53E+07	1,33e+10	2,05E+10
		$AN_{2.5}$	1,55E+07	1,93E+10	2,27E+10
		AE_0	3,80E+07	2,04E+10	2,27E+10
		$AE_{2.5}$	3,13E+07	1,54E+10	1,09E+10
3	2 years	AN_0	1,17E+08	1,55E+10	1,78E+10
		$AN_{2.5}$	4,01E+07	1,25E+10	1,50E+10
		AE_0	1,33e+08	1,94E+10	9,89E+09
		$AE_{2.5}$	1,47E+08	1,73E+10	1,44E+10
4	2 years	AN_0	6,79E+06	4,53E+09	1,78E+10
		$AN_{2.5}$	1,41E+07	1,10E+10	1,60E+10
		AE_0	2,10E+07	5,21E+09	1,98E+10
		$AE_{2.5}$	2,98E+07	1,05E+10	1,97E+10
5	1 month	AN_0	6,43e+06	3,47E+10	3,63E+10
		$AN_{2.5}$	7,63e+05	2,38E+10	2,46E+10
		AE_0	4,68E+06	1,53E+10	1,35E+10
		$AE_{2.5}$	2,00E+06	1,46E+10	1,91E+10
6	1 month	AN_0	1,06E+07	6,76E+09	2,51E+10
		$AN_{2.5}$	1,40E+07	1,07E+10	2,46E+10
		AE_0	1,63E+07	7,28E+09	7,28E+09
		$AE_{2.5}$	1,51E+07	2,96E+09	3,41E+09
7	1 month	AN_0	1,45E+06	2,64E+10	1,00E+10
		$AN_{2.5}$	1,63E+06	2,74E+10	1,26E+10
		AE_0	1,35E+06	1,18E+10	3,03e+09
		$AE_{2.5}$	1,65E+06	1,68E+10	2,87E+09
8	1 month	AN_0	9,86E+07	1,23E+10	2,58E+10
		$AN_{2.5}$	1,01E+08	9,01E+09	2,01E+10
		AE_0	2,73e+08	1,53e+10	1,77E+10
		$AE_{2.5}$	5,36E+08	1,16E+10	1,47E+10

Parameter	Numerator df	Denominator df	F test	P value
Intercept	1	6.000	22426.922	< 0.001
Atmosphere (anaerobic vs aerobic)	1	21.001	31.006	< 0.001
(In)direct processing	1	6.000	.896	.355
Storage time (1 month vs 2 years)	1	21.001	.828	.398
Atmosphere x Storage time	1	21.001	13.237	.002

Supplementary Table S3. Results from a general linear mixed model. Atmosphere had a main effect on the log CFU counts, whereas direct processing and storage time did not have main effects on the quantative bacterial outcomes. The atmosphere effect was modified by storage time, where differences between anaerobic and aerobic processing are larger for samples stored for 1 month compared to samples stored for 2 years.
