# Supplementary figures and images for: Anaerobic Feces Processing for Fecal Microbiota Transplantation Improves Viability of Obligate Anaerobes
Source: Microorganisms. 2023 Sep 5;11(9):2238. doi: 10.3390/microorganisms11092238 (PMC10535047; doi:10.3390/microorganisms11092238)

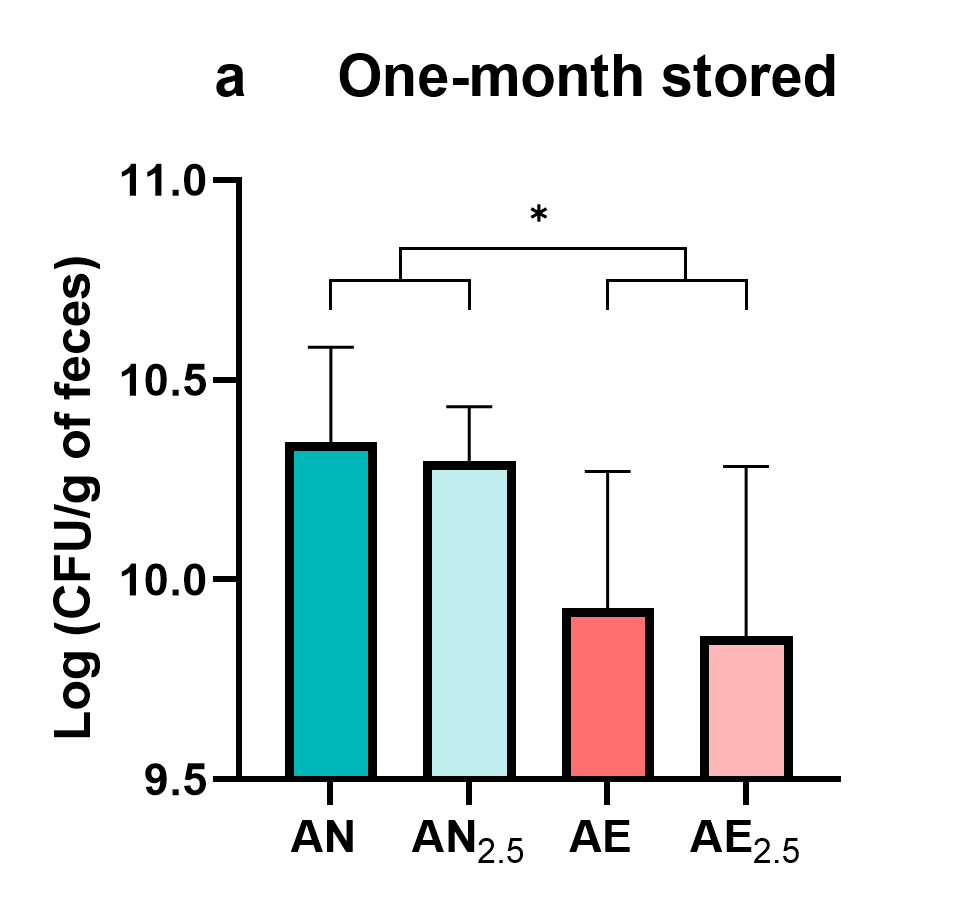

Supplement: Supplementary file 1 [file microorganisms-11-02238-s001.zip › Supplementary figure S1a.tif]

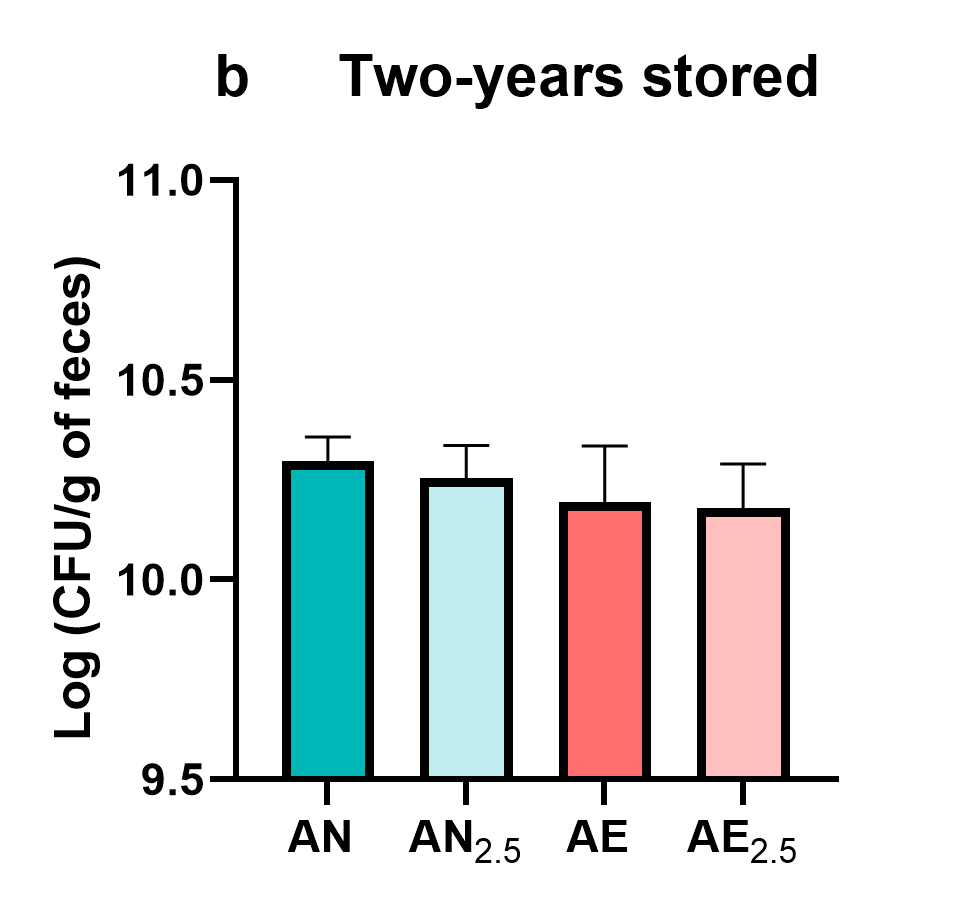

Supplement: Supplementary file 1 [file microorganisms-11-02238-s001.zip › Supplementary figure S1b.tif]

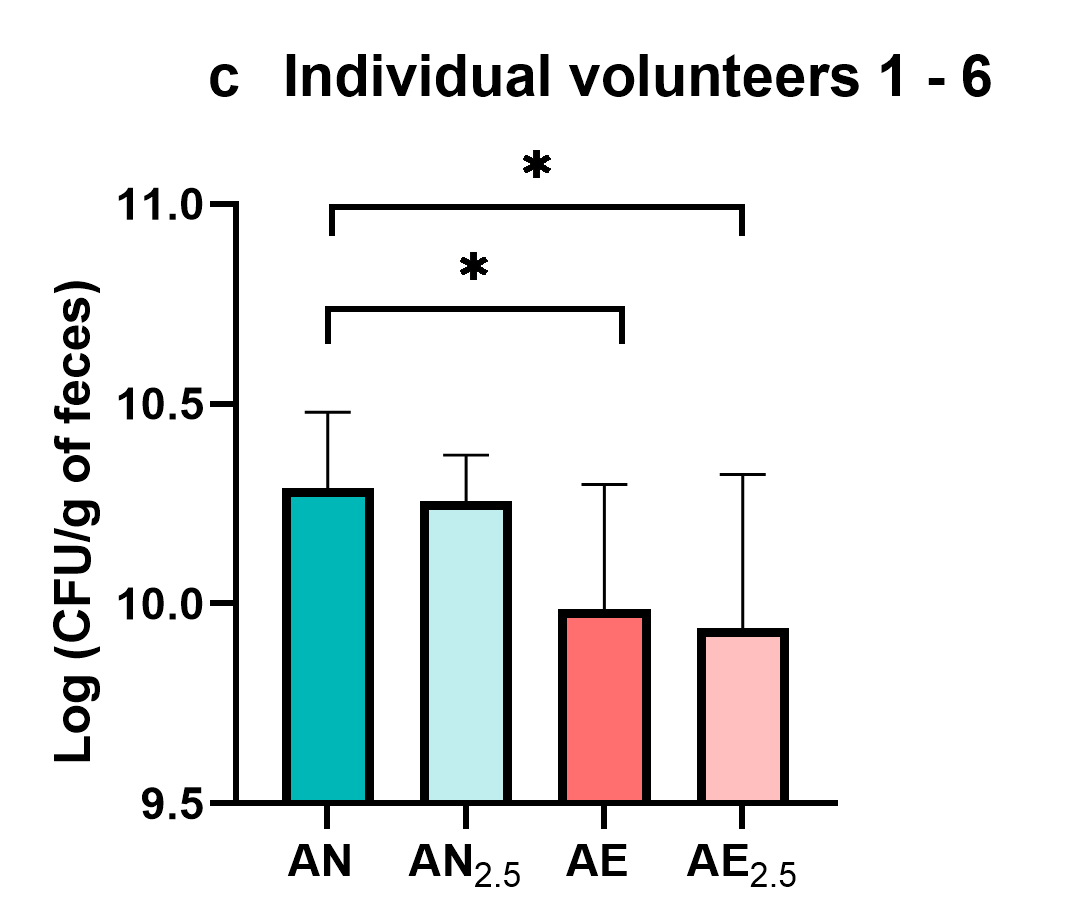

Supplement: Supplementary file 1 [file microorganisms-11-02238-s001.zip › Supplementary figure S1c.tif]
